# Broad-spectrum eye disease classification using a deep learning-based tailored software lens

**DOI:** 10.1371/journal.pone.0335419

**Published:** 2025-11-06

**Authors:** Celina Rieck, Luca Eisentraut, Ricardo Buettner

**Affiliations:** Chair of Hybrid Intelligence, Helmut-Schmidt-University/University of the Federal Armed Forces Hamburg, Hamburg, Germany; Deakin University, AUSTRALIA

## Abstract

The early and accurate classification of eye diseases is essential for preventing irreversible visual impairment. This task can be performed by deep learning approaches that automatically classify retinal fundus images according to potential illnesses. Despite notable advances in this field, the robust and methodologically rigorous classification of a broad range of eye diseases remains unsolved. This study addresses this issue by proposing a novel deep learning architecture that leverages specific features of retinal fundus images (e.g., image noise and importance of fine structures) using a tailored software lens to robustly diagnose a broad spectrum of illnesses at a high performance level. To validate this approach, the currently broadest peer-reviewed dataset of 16,242 images, comprising nine diseases and healthy samples, is chosen. Our novel architecture achieves a 5-fold cross-validated average balanced accuracy of 82.52 %, outperforming the baseline model (79.40 %) and setting a new benchmark. Our results demonstrate for the first time that high performance can be achieved for diagnosing a broad range of eye diseases based on retinal fundus images by leveraging their specific features. This approach has implications for clinical deployment, particularly in routine care settings, by enabling faster and more reliable screenings.

## Introduction

Eye diseases remain a major global health burden, with the World Health Organization estimating that over 2.2 billion people are affected by vision impairment, many of which are preventable or treatable through timely diagnosis and intervention [1]. Accurate classification of retinal conditions such as diabetic retinopathy, age-related macular degeneration (AMD), and glaucoma is therefore essential for effective early treatment and the prevention of irreversible vision loss [1,2]. However, diagnostic accuracy is often constrained by limited access to specialists and by the inherent subjectivity and variability in clinical assessments. Studies have shown that human misdiagnosis rates in ophthalmology can be substantial: In a large-scale diabetic retinopathy screening study, human graders achieved only 73.4 % sensitivity for referable disease and 62 % for diabetic macular edema, resulting in false-negative rates as high as 14.1 % for proliferative cases [3]. In neuro-ophthalmology, nearly 49 % of referrals were initially misdiagnosed, with 26 % of these leading to preventable patient harm due to delayed or incorrect treatment [2]. Moreover, manual interpretation of fundus images is time-intensive and lacks scalability for large-scale screening initiatives [4].

In contrast, systems based on deep learning (DL) have shown promising performance in processing images. Since being first developed in 1989 [5], their capability of classifying images and detecting objects has significantly increased [6,7]. In the last years, such systems have been transferred to medical applications on a large scale [8], outperforming human accuracy [9]. This ability makes DL approaches particularly useful in the field of ophthalmology, where diagnosis is largely based on images.

Consequently, many studies have utilized DL for the diagnosis of eye diseases [10–13]. For example, different stages of AMD have been differentiated [14], diabetic retinopathy diagnosed [11] and glaucoma detected [15]. The data basis used is almost exclusively fundus images, i.e. images of the fundus of the eye and the retina in particular. This large amount of existing studies can be divided into two groups: studies that use a peer-reviewed dataset [14,16,17] and studies that cover a broad range of eye diseases [18,19]. A **relevant research gap** emerges: no approach exists to date that covers all relevant eye diseases using a peer-reviewed dataset. This gap is critical, since such an approach is relevant to prevent misclassifications. For clinical use, the high-quality methodological development and validation reached by employing a peer-reviewed dataset is also essential to ensure robust and reproducible performance.

This study aims to **address this gap** by proposing an innovative DL architecture that is able to robustly classify a broad range of relevant eye diseases. To achieve this, instead of relying on a more complex architecture, the specific characteristics of the images to be classified are utilized through the design of the architecture. Retina fundus images typically exhibit image noise, additionally, recurring high-frequency artifacts can potentially lead to overfitting [20–23]. From a medical point of view, fine structures such as vessels must be considered with particular accuracy [24]. In order to take these characteristics into account, our study uses the ideally suited **Gaussian filter as a software lens** to reduce noise and high-frequency artefacts and to emphasize fine structures [25,26]. In line with the aim of our study, we train and validate our approach employing, to the best of our knowledge, the most comprehensive peer-reviewed dataset (nine eye diseases and healthy cases, 16,242 images [4]). Using a 5-fold cross-validation, our model achieves an average balanced accuracy of 82.52 %, setting a **new benchmark**. This is an important step towards high-performance, DL-based diagnostics of eye diseases, which will enable broad screening initiatives, and thus a significant improvement in the health of society as a whole. The contributions of our work are:

1. Development of a novel DL architecture that leverages characteristics of retinal fundus images through Gaussian filtering.
2. Establishment of a new benchmark in this domain by achieving an average balanced accuracy of 82.57 % using a 5-fold cross-validation.
3. Demonstration that architectures tailored to the specific problem characteristics can significantly enhance model performance.

The structure of this work is as follows: First, the *Related work* section provides an overview of the state-of-the-art in this research area, before our *Methodology*, including the model architecture, innovation, and training process, is presented. Afterwards, we outline the evaluation *Results*, followed by a *Discussion* of their implications. Finally, limitations and potential directions for future work are presented.

## Related work

### Relevant eye diseases

This study classifies a broad range of eye diseases and healthy samples based on retinal fundus images. To this end, it uses the currently most comprehensive peer-reviewed dataset on retinal fundus images [4], which contains nine clinical pictures and is therefore extremely diverse. These diseases cover most of the most common diseases and are briefly characterized below:

- *CSC – Central Serous Chorioretinopathy*: Often affecting men and starting to occur from early adulthood on, *CSC* is identified by a visible retina separation caused by a leakage in the retinal pigment epithelium and a fluid accumulation underneath the retina [27].
- *DR – Diabetic Retinopathy*: A progressive disease in which small vessels of the retina are damaged by diabetes, which is manifested by various smaller lesions, e.g., microaneurysms [24].
- *DE – Disc Edema*: If the optic nerve head swells, its edge appears as a raised characterization. This can be a sign of neurological diseases, e.g. inflammation, which can be of a more serious nature [28].
- *GL – Glaucoma*: Visible in fundus images, inter alia, as thinning of the optic nerve center, glaucoma causes irreversible damage to the optic nerve. This is one of the most common causes of blindness [29].
- *MS – Macular Scar*: A collective term for all types of scarring in the macula. These can be caused by inflammation or other diseases and manifest themselves as color changes or other irregularities [30].
- *MY – Myopia*: A very common limitation in which so-called pathological myopia occurs due to elongation of the eyeball. This can potentially lead to retinal tears, for example, which may previously show up as structural changes in fundus images [31].
- *PT – Pterygium*: If tissue grows from the conjunctiva towards the cornea, this often occurs in the shape of a wing, which is called pterygium. This disease is visible not at the retina fundus, but on the front surface of the eye [32].
- *RD – Retinal Detachment*: An often sudden clinical condition in which the sensory retina detaches. Must be treated as a medical emergency and is manifested by gray/wavy areas in fundus images [33].
- *RP – Retinitis Pigmentosa*: A hereditary disease in which the retina slowly degenerates and can lead to considerable impairment. In fundus images, this is usually shown by dark clumps [34].

In addition to these diseases, the dataset also contains healthy samples. On retinal fundus images, a healthy retina is characterized by a clear representation of the retinal structure, i.e., the blood vessels and the macula, without any forms of swelling or lesions. In terms of color, the image appears relatively uniform, with a reddish-orange hue due to the reflection of light.

### Eye disease detection

Early detection of eye diseases is key to preventing permanent vision loss and starting treatment in time [1]. Common conditions such as diabetic retinopathy, glaucoma, cataract, and AMD can cause serious damage to the eye if not detected early, as they often begin without noticeable symptoms, which makes regular eye examination important [1,2]. Color fundus photography is a widely used method that takes detailed images of the retina. It helps physicians spot early symptoms of diseases, thereby reducing the risk of serious visual impairment. However, viewing and analyzing these images by hand takes time and requires trained eye specialists, which can be a challenge in daily healthcare settings [1,4]. This not only makes broad screening of the population impossible in situations where images could be taken easily, but no physician is available for assessment (e.g., at the optician’s). Additionally, even if a doctor is available, the assessment of such images is prone to error due to the human component [3].

This is where DL can play a valuable role. DL-based systems can automatically review large numbers of fundus images and help identify possible eye diseases. These tools are currently mainly not intended to replace doctors, but to support them, for example, by helping opticians or general practitioners spot unusual cases and refer patients to eye specialists when needed. This could make eye care more efficient and allow for faster diagnosis for patients [2–4]. However, such an approach only reaches its full potential when a wide range of diseases can be identified, preventing misclassifications on the one hand and allowing more differentiated information to be provided on the other. This requires appropriately performing approaches.

### Evolution of modern convolutional neural networks

Deep Learning mainly employs convolutional neural networks (CNNs), that are information processing paradigms. They can independently recognize patterns and develop filters to extract features that are used for classification in a combined step, excelling in classifying images [5,6]. Today’s CNNs are much more capable of more complex recognition tasks [7]. As a result, they surpass human quality in more and more domains such as industrial quality control [26,35,36], medical imaging [8,9] or precision agriculture [37,38].

With their novel architecture, AlexNet, Krizhevsky et al. [39] achieved the first major breakthrough in solving more complex tasks and created the first model ever to win the ImageNet competition. Over the next few years until today, VGGNet [40], GoogleNet (Inception Models) [41], and ResNet [42] were the architectures that were able to build on the success and achieve even better accuracies. Modern CNNs have been enhanced by architectural developments such as hybrid architectures [43,44] or triplet architectures [45]. But also, improvements in pre-processing methods and corresponding filters have led to better results [26,46].

### Deep learning detection of eye diseases

Due to the high relevance of the problem, a large number of studies have recently used deep learning-based methods for the detection of eye diseases. This has resulted in two groups: those that work solely on the basis of images and those that also incorporate other data in addition to images and are therefore hybrid/multimodal [47,49].

The latter group includes, for example, [47], which diagnoses seven eye diseases based on fundus and OCT images (evaluated by a CNN), clinical risk factors (evaluated by a GNN), and texts from medical reports (evaluated by an LLM). When distinguishing between seven diagnoses, the RetinalOCT system presented can achieve an accuracy of 98 %, which significantly outperforms pure CNN-based systems. Ma et al. [48] also rely on a multimodal approach, distinguishing between 50 diseases based on a combination of text-based input (doctor-patient dialogues) and optical images (e.g., slit lamp images). Using ChatGPT-powered AI, an accuracy of 79.6 % (internal) and 81.1 % (external) can be achieved. Even though such multimodal approaches are useful for certain applications, image-based input information is almost always necessary for this purpose, which is why it must also be optimized. In addition, purely image-based models can be easy to deploy in certain domains, such as broad screening.

For this reasons, current research is predominantly focused on purely image-based approaches. Although numerous studies have explored a range of CNN architectures for the automated detection and classification of eye diseases, a key limitation persists, as shown in Table 1: **no relevant study** utilizes a **peer-reviewed dataset and** covers a **broad range of diseases**.

**Table 1:** Overview of prior studies on automated eye disease detection. The comparison highlights whether each work utilizes on a peer-reviewed dataset and whether it addressed a broad spectrum of eye diseases. Most studies do not fulfill each one of both aspects, while only a few cover one of the two. The proposed study uniquely combines both peer-reviewed data and broad disease coverage.

Authors	Peer-reviewed Dataset	Broad Disease Coverage
Abbas et al. [10]	✗	✗
Bernabé et al. [11]	✗	✗
Hossain et al. [12]	✗	✗
Oh et al. [13]	✗	✗
Xu et al. [15]	✗	✗
Wahab Sait and Rahaman [50]	✗	✗
Babaqi et al. [51]	✗	✗
Biswas et al. [52]	n/a	✗
Glaret S. and Muthukannan [53]	✗	✗
He et al. [18]	✗	✓
Al-Fahdawi et al. [19]	✗	✓
Grassmann et al. [14]	✓	✗
Tomar et al. [16]	✓	✗
Chea/Nam [17]	✓	✗
This study	✓	✓

Some approaches rely on non peer-reviewed datasets, limiting the transparency, reproducibility, and clinical transferability of their results [10–13]. Additionally, existing work remains mainly constrained to low-scale multiclass classification tasks; typically involving only 2-5 disease categories and not covering a broad range of diseases [14,16]. This narrow focus, combined with inconsistent dataset quality, poses challenges for building robust and generalizable models suitable for real-world clinical use. To address these issues, our study utilizes a comprehensive, peer-reviewed dataset comprising a healthy class and nine disease types.

#### Studies utilizing a non-peer-reviewed dataset.

The following studies exemplify how various architectures, datasets, and diagnostic targets have been leveraged to achieve high-performance binary classification in ophthalmological and neurological imaging contexts. Abbas [10] propose "Glaucoma-Deep", a DL framework combining unsupervised feature extraction via a CNN, discriminative feature selection using a deep belief network, and a softmax classifier. The model achieves a cross-validated accuracy of 99 % across a non-peer-reviewed dataset of 1,200 retinal images. Bernabé et al. [11] develop a CNN-based classifier to differentiate between diabetic retinopathy and glaucoma. Validated via K-fold cross-validation, the model achieves 99.89 % classification accuracy on a non-reviewed dataset of 565 images. Hossain et al. [12] develop an automated cataract detection system using a modified ResNet-50 architecture, trained directly on fundus images without prior preprocessing, achieving an accuracy of 95.77 % on a non-reviewed 5,718 image dataset with a fixed split. Oh et al. [13] utilize an even larger dataset of 13,271 images (non-peer-reviewed). The authors address early diabetic retinopathy detection using ultra-wide-field fundus images, which capture up to 200^°^ of the retina. By comparing 7-field images with optic disc-centered views, the system achieved a cross-validated AUC of 0.9150. Xu et al. [15] introduce the so-called "Transfer Induced Attention Network", a transfer learning-based model for glaucoma detection. The architecture utilized ophthalmic source domain data and channel-wise attention with maximum mean discrepancy to enhance feature transfer. TIA-Net achieved accuracies of 85.7 % and 76.6 % on two non-reviewed clinical datasets.

In 2023, Wahab Sait and Rahaman [50] developed a lightweight model combining denoising autoencoders and single-shot detection for feature extraction. Evaluated on the ODIR and EDC datasets (7,000 images, neither peer-reviewed nor cross-validated), the system achieved 99.4 % accuracy. Babaqi et al. [51] use transfer learning to classify fundus images into four categories: normal, diabetic retinopathy, glaucoma, and cataract. Their fine-tuned pre-trained CNN model, trained on 4,200 labeled images (non-reviewed), achieves a cross-validated 94 % accuracy. Biswas et al. [52] proposed an AI-based system using CNNs to classify and localize eye diseases such as diabetic retinopathy, glaucoma, and cataract from fundus images. Their model achieved an accuracy of up to 93 %, but lacks cross-validation. Glaret subin and Muthukannan [53] propose a CNN optimized via the flower pollination algorithm for classifying a five-task problem, consisting of diabetic retinopathy, glaucoma, AMD, and cataracts. Their model, incorporating entropy-based preprocessing and support vector machines, achieves a cross-validated accuracy of 95.27 % on a non-peer-reviewed dataset.

Besides those studies that neither utilize a peer-reviewed dataset nor cover a broad range of eye diseases, two works address a broader range while still employing such a dataset. He et al. [18] introduced DCNet, a dense correlation CNN designed for multi-label classification of ocular diseases using spatial correlations between left and right fundus images. The network featured a spatial correlation module and patient-level feature fusion and is evaluated on 3,500 non-reviewed images. Al-Fahdawi et al. [19] introduce Fundus-DeepNet, a novel multi-label DL framework designed to detect eight ocular diseases from fundus images. The model features an extensive preprocessing pipeline and a hybrid architecture integrating HRNet, attention mechanisms, SENet blocks, and a discriminative restricted Boltzmann machine. They also perform feature-level fusion of left and right eye images. The system delivered AUC scores of 99.76 % (off-site) and 99.86 % (on-site), and F1-Scores exceeding 88 % on a non-reviewed dataset of 10,000 images.

#### Studies utilizing a peer-reviewed dataset.

Three studies utilize a peer-reviewed dataset, however, they do not cover a broad range of eye diseases. Grassmann et al. [14] develop a DL model only for AMD classification using the AREDS severity scale. Even though this makes it a multiclass problem, this approach does not cover a broad range of eye diseases, as mainly stages AMD severity are classified. An ensemble of six CNNs (Inception, ResNet, VGG) was trained on over 120,000 fundus images. The system achieved a weighted kappa of 0.92, correctly identifying 94.3 % of healthy cases and 84.2 % of AMD cases on an external dataset. Tomar et al. [16] propose a DL model for diabetic retinopathy detection. The model, evaluated on the IDRiD dataset (518 images), demonstrated high classification accuracy and was particularly effective in grading DR severity level; however, no cross-validation was employed. Finally, Chea/Nam [17] focus on classifying fundus images using DL to detect eye diseases such as diabetic retinopathy, glaucoma, and AMD. Their approach combined optimized residual deep neural networks with advanced image preprocessing techniques, including iso-luminance plane histogram equalization and data augmentation. Their model achieves an average accuracy of 85.79 % for AMD detection (cross-validated, 2,335 samples), but does not cover a broad range of eye diseases.

## Methodology

We utilize both a baseline model and an innovative model architecture that differ by applying a Gaussian filter as a software lens specifically tailored to the problem domain. For both models, first, a ResNet-50 architecture is imported as a base model with ImageNet weights and enriched with additional custom layers to create a transfer learning model. A 5-fold cross-validation is then carried out with the model and the dataset used in order to achieve valid and robust results. For each training split of the cross-validation, the best hyperparameters are first searched for, and the split used is enhanced with data augmentation. Before the entire model is trained, the custom layers are first trained individually, then, the entire model is trained at a lower learning rate in order to comply with the transfer learning method. Finally, the performance is evaluated using images from a previously unused data portion.

### Model architecture

**Fig 1:**
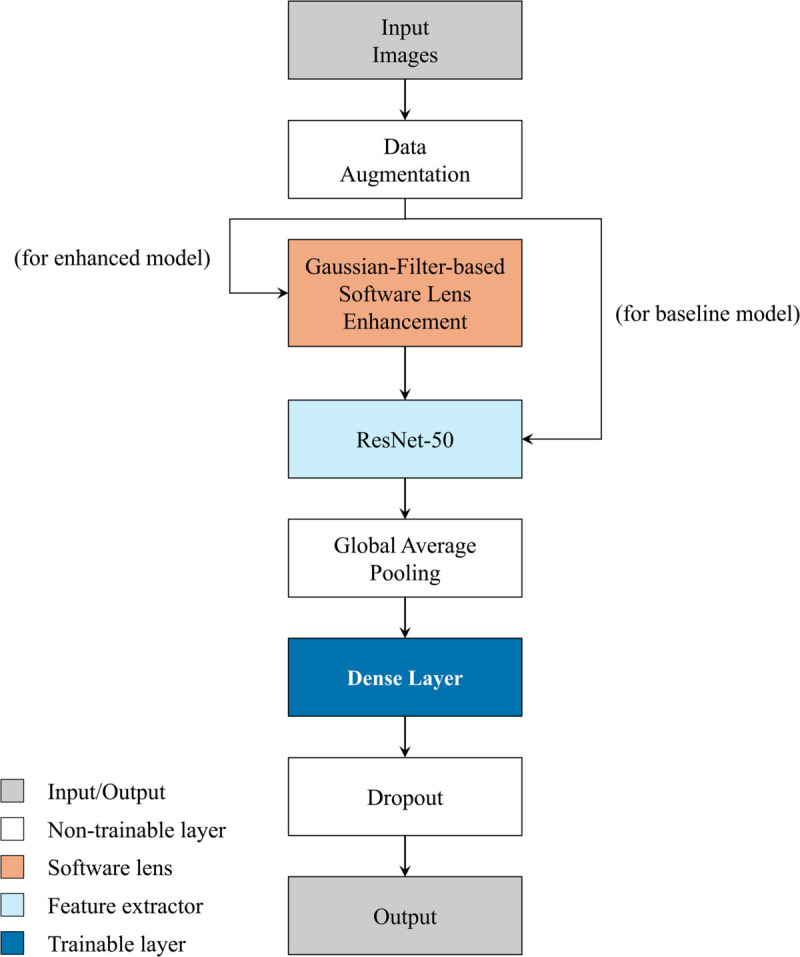
Pipeline of our proposed model. Input images are augmented. In the enhanced variant a Gaussian-filter “software lens” is applied before ResNet-50, while the baseline model skips this step. The extracted features are globally averaged and passed to a dense layer, dropout layer, and the classification head. Color legend: gray = input/output, white = non-trainable, orange = software lens, light blue = feature extractor, dark blue = trainable dense.

Our baseline model consists of the following layers: input layer, data augmentation (rotation, zoom, translation), ResNet-50 backbone, global average pooling layer, dense, dropout, and output layer; visualized in Fig 1.

The input shape of our model is 150,150,3. The backbone of our model was introduced by He et al. [42]. ResNet-50 is comparable to VGG16 and VGG19, but with 50 layers and an additional identity mapping capability. If the current layer is not needed, this identity allows it to be bypassed, which reduces overfitting problems [42]. The top layer of the backbone is excluded, as it is specific to the dataset used to train the respective model. All layers of the ResNet-50 backbone are initially frozen, such that only the custom layers are trainable. During fine-tuning, the last 20 layers are later unfrozen.

For DL models, larger datasets lead to better accuracies. However, the problem with large datasets is that they require a lot of effort to acquire and label the data. For this reason, data augmentation is used to generate good DL models even with smaller datasets [54]. For the model used, we utilize selected data augmentation layers from the Keras library. These layers modify an image with given parameters before feeding it into the model in order to increase the variance of the training data. Three augmentation layers are used, which randomly rotate the image, perform a random translation, or apply a random zoom. The augmentation layers are placed before the backbone, while the additional custom layers serve as classification heads and are placed after the backbone. For the enhanced model, a filter is inserted between the data augmentation and the backbone. For the custom layer, again, Keras library is used. First, a 2D global average pooling layer is added, which is followed by a dense layer. This dense layer uses a rectified linear unit activation function. A dropout layer is then used to prevent overfitting and a dense layer with a sigmoid activation function is used as the final classification layer. This output layer consists of 10 softmax units corresponding to the classes in the dataset. The model uses an Adam optimizer with the AMSGrad extension and is compiled with a cross-entropy loss function.

### Gaussian-filter-based software lens

In this work, we employed a Gaussian filter as a software lens to enhance the accuracy and robustness of the classifier, whereas this filter suits the specific characteristics of our problem domain and has proven its performance-enhancing character in medical tasks [9]. The Gaussian filter performs a smoothing of the respective image by convolving each pixel with a Gaussian kernel [25]. Initially, the model was trained and evaluated without any preprocessing, as previously described. In a second run, the filter was applied to each image prior to being passed to the model. To ensure a fair comparison between both approaches, with and without the preprocessing step, the training and evaluation procedures were kept identical across both runs. The Gaussian filter is a low-pass filter that attenuates high-frequency noise by convolving the input image with a two-dimensional Gaussian function [25]. This function is defined by a bell-shaped curve:

$$G(x,y)=\frac{1}{2\pi \sigma^{2}}e^{-\frac{x^{2}+y^{2}}{2\sigma^{2}}},$$ (1)

where (*x*,*y*) represent the spatial coordinates of each pixel and *σ* denotes the standard deviation of the Gaussian distribution, which governs the extent of smoothing. The higher values of *σ* result in broader smoothing kernels that assign greater weight to more distant neighboring pixels, while the lower values lead to more localized averaging [25]. In practice, each pixel’s new value is computed as a weighted average of its surrounding pixels within a kernel *k*, with the weights derived from the Gaussian function. In this study, we employ a 5×5 kernel, and the value of *σ* is determined according to OpenCV’s empirical formula [55]:

$$\sigma =0.3\cdot \left(\frac{k-1}{2}-1\right)+0.8,$$ (2)

which yields σ=1.1 for *k* = 5. This configuration enables effective noise suppression while preserving edge integrity, outperforming simpler averaging filters in maintaining diagnostically relevant features. The filter thus contributes to image quality enhancement in a manner that is well-suited for downstream classification tasks.

### Training process

**Fig 2:**
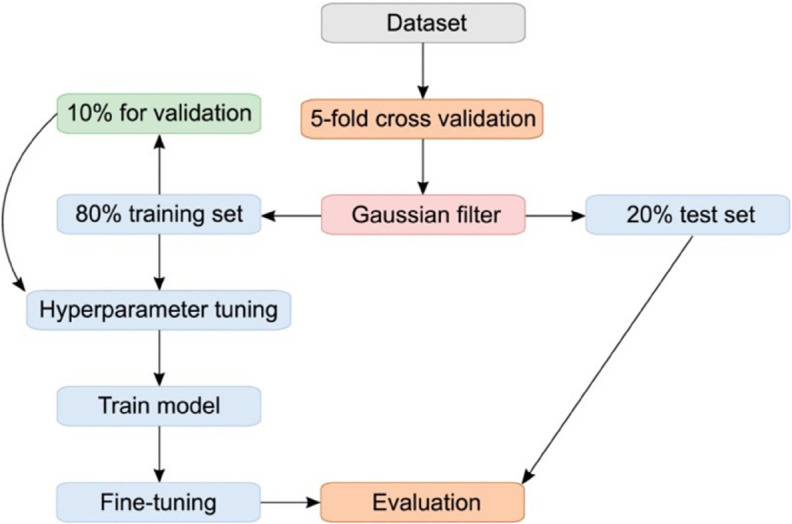
Training workflow used in this study. The dataset is split into training, validation, and test sets, with Gaussian filtering applied as a preprocessing step in the enhanced model. The training set is used for hyperparameter tuning, model training, and fine-tuning, while evaluation is performed on the test set using 5-fold cross-validation.

The training process is shown in Fig 2. It is carried out twice, with the difference that the images are preprocessed with the innovative filter in the second run.

To ensure the randomness of the approach and data splitting, we set a random seed of 42. This seed was used for all relevant libraries to ensure complete reproducibility. Before the images of the selected dataset can be fed into the model, they are preprocessed using the built-in ImageDataGenerator function of Keras. The standard function flow_from_dataframe was used here, which loads images based on labels and paths. The generator automatically loads, shuffles, and batches the data. The images are randomly reordered, and subsequently resized to 150 x 150 pixels. This resizing is necessary because the images have different input sizes, which are also too large for processing in the pipeline. All images are loaded in the three RGB color channels, so the input dimension is 150,150,3. The images are also normalized and scaled according to the ImageNet pretraining of our backbone to be in the same value range as the pretraining images. Then, the images are loaded with a batch size of 32 by the ImageDataGenerator instance. This preprocessing is performed collectively before the data is split. In addition, the individual class weights are used in the model during training in order to obtain weighted results. The weight *w*_*i*_ of each class *i* is computed as

$$w_{i}=\frac{N}{2\cdot n_{i}},$$ (3)

where *N* denotes the total number of samples, *n*_*i*_ the number of samples per class. Since an imbalanced dataset is used in this study, this ensures that underrepresented classes have a proportionally higher importance during training. The scaling factor used stabilizes the overall loss magnitude. The resulting weights *w*_*i*_ are passed to the Keras training routine via the class_weight argument. Since the loss contribution of each sample is multiplied by this weight *w*_*i*_, it ensures that minority classes have a stronger influence on the gradient updates.

Afterwards, a 5-fold cross-validation splitting is performed, which divides the data into 80 % training split and 20 % testing split. Another 10 % of the images are then separated from the training split as a validation split. The random state was set to 42 to ensure the reproducibility of the results.

The **first training stage** is the hyperparameter tuning. We use the random search tuner of the Keras library with the aim of minimizing validation loss. The tuner is used to optimize the parameters of the model and uses the validation split for evaluation. It utilizes the Adam (AMSGrad) optimizer and a sparse categorical cross-entropy loss, the ResNet-50 backbone remains frozen during the training stage. The trials are undertaken independent for each fold to prevent data leakage. The tuner has a maximum number of 20 trials for each fold. For the tuner, we use additional early stopping with a patience of ten, which terminates the trial if the validation loss does not improve for ten epochs. Each trial is conducted with 40 epochs. The parameters that are optimized by the tuner and the corresponding values are shown in Table 2.

**Table 2:** Hyperparameters used during hyperparameter tuning. Data augmentation settings (rotation, zoom, translation) are varied within the defined ranges and with the defined steps, while model parameters (dense layer size, dropout rate, learning rate) were tested across the specified values.

Hyperparameter	Minimum Value	Maximum Value	Step
Random Rotation	0.05	0.2	0.05
Random Zoom	0.05	0.2	0.05
Random Translation Height	0.05	0.2	0.05
Random Translation Width	0.05	0.2	0.05
Dense Layer Units	64	128	32
Dropout Rate	0	0.3	0.1
Learning Rate	10^−5^, 10^−4^, 10^−3^		

The **second training stage** is the training of the custom layers. For each fold, the best parameters of the hyperparameter tuning are utilized for this stage. Here, only the custom layers are trained, whereas the layers of the backbone still remain frozen. The model is trained with a maximum of 100 epochs and a patience of ten; the optimizer and loss function remain identical to the first stage. After finishing the training process, the model of the epoch with the best results is automatically restored and passed forward into the next stage. In the **third and final training stage**, fine-tuning is performed. The top 20 layers of the utilized ResNet-50 backbone are unfrozen during this training stage and are therefore fine-tuned. The learning rate is set to 10^−5^ so that the model does not suffer from overfitting problems. The model is fine-tuned for 30 further epochs with a patience of ten using callbacks for validation loss. Again, the model of the best epoch is automatically restored and used for evaluation after the termination of the fine-tuning process.

### Evaluation metrics

To evaluate and interpret the model’s performance, we employ the following performance indicators as implemented in scikit-learn [56]:

**Accuracy** indicates the ratio of correctly predicted instances (true positives, TP, and true negatives, TN) to the total number of predictions (TP and TN, additionally, false positives, FP, and false negatives, FN) [57]:

$$\text{Accuracy}=\frac{TP+TN}{TP+TN+FP+FN}$$ (4)

**Balanced Accuracy** (bal. acc.) calculates the average recall over all classes, providing a more robust measure for imbalanced datasets [58]. *C* denotes the number of classes, *i* the respective class.

$$\text{Bal. Acc.}=\frac{1}{C}\sum_{i=1}^{C}\frac{TP_{i}}{TP_{i}+FN_{i}}$$ (5)

**Recall** reflects the ratio of TP to the total samples in this class [59], with *n*_*i*_ denoting samples per class and *N* the overall number of samples in the task.

$$\text{Recall}=\sum_{i=1}^{C}\frac{n_{i}}{N}\cdot \frac{TP_{i}}{TP_{i}+FN_{i}}$$ (6)

**Specificity** denotes the proportion of actual negative instances correctly identified as such [60].

$$\text{Specificity}=\sum_{i=1}^{C}\frac{n_{i}}{N}\cdot \frac{TN_{i}}{TN_{i}+FP_{i}}$$ (7)

**Precision** denotes how many of the samples assigned to a class are correct, highlighting the ratio of how many instances were incorrectly assigned to a class [59].

$$\text{Precision}=\sum_{i=1}^{C}\frac{n_{i}}{N}\cdot \frac{TP_{i}}{TP_{i}+FP_{i}}$$ (8)

**Negative Predictive Value** (NPV) refers to the proportion of TN among all predicted negatives [60].

$$\text{NPV}=\sum_{i=1}^{C}\frac{n_{i}}{N}\cdot \frac{TN_{i}}{TN_{i}+FN_{i}}$$ (9)

**F1-Score** is the harmonic mean of precision and recall [57]. It is particularly valuable in scenarios where both false positives and false negatives carry significant consequences.

$$\text{F1-Score}=2\cdot \frac{\text{Precision}\cdot \text{Recall}}{\text{Precision}+\text{Recall}}$$ (10)

**Cohen’s Kappa (κ)** evaluates the agreement between predicted and true labels, taking into account the agreement by chance [61]. Here, *P*_*o*_ represents the observed agreement, and *P*_*e*_ the expected agreement by chance.

$$\kappa =\frac{P_{o}-P_{e}}{1-P_{e}}$$ (11)

### Dataset

This study utilizes the *Eye Disease Image Dataset* introduced by Sharmin et al. [4], which provides a diverse collection of high-quality images of various eye diseases. The dataset contains a total of 16,242 images, collected over a period of eight months from Anawara Hamida Eye Hospital and B.N.S.B. Zahurul Haque Eye Hospital, located in the Faridpur district of Bangladesh. The images were captured using Topcon TRC-50DX and TL-211 fundus cameras connected to Nikon DSLR cameras, with image resolutions ranging from 2,004×1,690 to 5,600×3,728 pixels, and stored in .jpg format. The dataset is publicly available through the Mendeley Data Repository.

**Fig 3:**
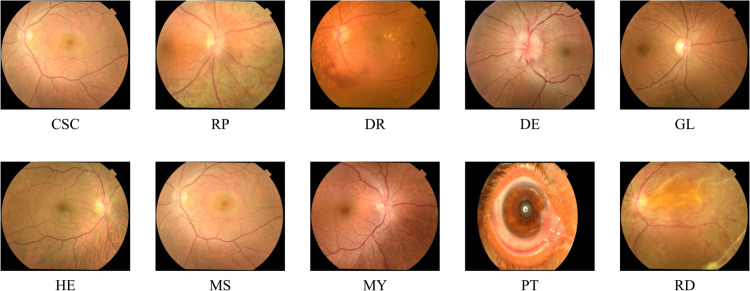
Representative fundus images of the dataset, that illustrate the 10 diagnostic categories: Central Serous Chorioretinopathy (CSC), Retinitis Pigmentosa (RP), Diabetic Retinopathy (DR), Disc Edema (DE), Glaucoma (GL), Healthy (HE), Macular Scar (MS), Myopia (MY), Pterygium (PT), and Retinal Detachment (RD) [4].

The dataset is divided into ten classes, as shown in Table 3 and illustrated in Fig 3. Specifically, it includes 606 samples of *CSC*, 834 samples of *RP*, 3,444 of *DR*, 762 of *DE* and 2,880 samples of *GL*. Additionally, the data contains 2,676 images of *HE*, 1,937 of patients suffering from *MS*, 2,251 of patients suffering from *MY*, 102 of *PT* and 750 from *RD*. Each image in the dataset has been labeled and verified by medical experts.

**Table 3:** Class distribution of the dataset. Each line lists the abbreviation, full disease name, and number of samples. The dataset covers 10 categories with varying representation. It ranges from rare cases such as *Pterygium* (102 samples) to highly represented classes such as *Diabetic Retinopathy* (3,444 samples).

Abbreviation	Full Disease Name	Samples
CSC	*Central Serous Chorioretinopathy*	606
RP	*Retinitis Pigmentosa*	834
DR	*Diabetic Retinopathy*	3,444
DE	*Disc Edema*	762
GL	*Glaucoma*	2,880
HE	*Healthy*	2,676
MS	*Macular Scar*	1,937
MY	*Myopia*	2,251
PT	*Pterygium*	102
RD	*Retinal Detachment*	750

## Results

First, the baseline model, which is based solely on a ResNet-50 backbone, was evaluated. The results are shown in Table 4. The average accuracy is 72.40 %, the average bal. acc. is 76.88 %. The evaluation shows an average recall of 72.40 % and an average specificity of 96.77 %. The average precision is 72.40 %, and the NPV averages 96.78 %. The F1 score and kappa, also averaged, are 72.40 % and 67.75 %, respectively.

**Table 4:** Performance of the baseline model across five cross-validation folds (I-V). Reported metrics are Accuracy, Balanced Accuracy, Recall (TPR), Specificity (TNR), Precision (PPV), NPV, F1-Score, and Cohen’s Kappa. The averages of each metric are shown in the last column. Results indicate consistent performance across folds, with average accuracy of 72.4 % and strong specificity (96.8 %). This reflects robustness of the model but also highlights limited sensitivity compared to specificity.

Metric	I	II	III	IV	V	Avg.
Accuracy	0.7313	0.7439	0.6983	0.7263	0.7201	0.7240
Balanced Accuracy	0.7775	0.7798	0.7556	0.7666	0.7644	0.7688
Recall (TPR)	0.7313	0.7439	0.6983	0.7263	0.7201	0.7240
Specificity (TNR)	0.9684	0.9698	0.9650	0.9680	0.9673	0.9677
Precision (PPV)	0.7313	0.7439	0.6983	0.7263	0.7201	0.7240
NPV	0.9683	0.9702	0.9648	0.9682	0.9671	0.9678
F1-Score	0.7313	0.7439	0.6983	0.7263	0.7201	0.7240
Kappa	0.6857	0.7001	0.6486	0.6801	0.6730	0.6775

Table 5 presents the performance metrics of the proposed model. It achieves an avg. accuracy of 77.26 %, with individual folds ranging from 75.62 % (Fold V) to 78.61 % (Fold I). The bal. acc. averaged 82.52 %, with fold-level values spanning from 81.76 % to 83.57 %. The Recall and the Precision both averaged 77.26 %. The model’s ability to correctly reject negative cases was particularly strong: the specificity reached an average of 97.35 %. Similarly, the NPV mirrored this robustness, also averaging 97.35 %, with a minimum of 97.12 % (Fold V) and a maximum of 97.48 % (Fold I). The F1-Score was 77.26 % on average, confirming a balanced trade-off between Precision and Recall, with values ranging from 75.62 % (Fold V) to 78.61 % (Fold I). Finally, Cohen’s Kappa coefficient averaged 73.52 %, with the lowest value being 71.63 % (Fold V) and the highest 75.04 % (Fold I), indicating consistency across folds.

**Table 5:** Performance of the proposed model with Gaussian filtering across five cross-validation folds (I-V). Metrics include Accuracy, Balanced Accuracy, Recall (TPR), Specificity (TNR), Precision (PPV), NPV, F1-Score, and Cohen’s Kappa, with averages in the final column. Compared to the baseline (Table 4), the model achieves higher average accuracy (77.3 % vs. 72.4 %) and balanced accuracy (82.5 % vs. 76.9 %), while maintaining very high specificity (97.4 %). This indicates overall improved classification performance and better handling of class imbalance.

Metric	I	II	III	IV	V	Avg.
Accuracy	0.7861	0.7818	0.7805	0.7586	0.7562	0.7726
Balanced Accuracy	0.8271	0.8357	0.8249	0.8206	0.8176	0.8252
Recall (TPR)	0.7861	0.7818	0.7805	0.7586	0.7562	0.7726
Specificity (TNR)	0.9750	0.9746	0.9745	0.9720	0.9717	0.9735
Precision (PPV)	0.7861	0.7818	0.7805	0.7586	0.7562	0.7726
NPV	0.9748	0.9742	0.9742	0.9717	0.9712	0.9735
F1-Score	0.7861	0.7818	0.7805	0.7586	0.7562	0.7726
Kappa	0.7504	0.7458	0.7443	0.7192	0.7163	0.7352

In addition to evaluating the baseline model and the proposed model with Gaussian filtering, we performed a preliminary benchmarking analysis to compare our chosen backbone with other backbones. For efficiency reasons, this preliminary benchmarking was performed with only 10 trials during hyperparameter search and without fine-tuning. Gaussian filtering was used. The results of this analysis (average balanced accuracy over five folds) are shown in the following Table 6. In addition to ResNet-50, Densenet121 and EfficientNetV2 were used as recent models. DenseNet121 achieved the lowest performance with an average balanced accuracy of 79.67 %, EfficientNetV2 achieved an average value of 80.73 %, and the ResNet-50 backbone used in our main analysis achieved the best value with 81.90 %.

**Table 6:** Preliminary benchmarking of different CNN backbones on the dataset. Results report the average balanced accuracy across five cross-validation folds with 10 hyperparameter tuning trials and without fine-tuning. Among the tested architectures, ResNet-50 achieved the best performance (81.90 %).

Model	DenseNet121	EfficientNetV2	ResNet-50
Average Balanced Accuracy	79.67 %	80.73 %	**81.90 %**

**Fig 4:**
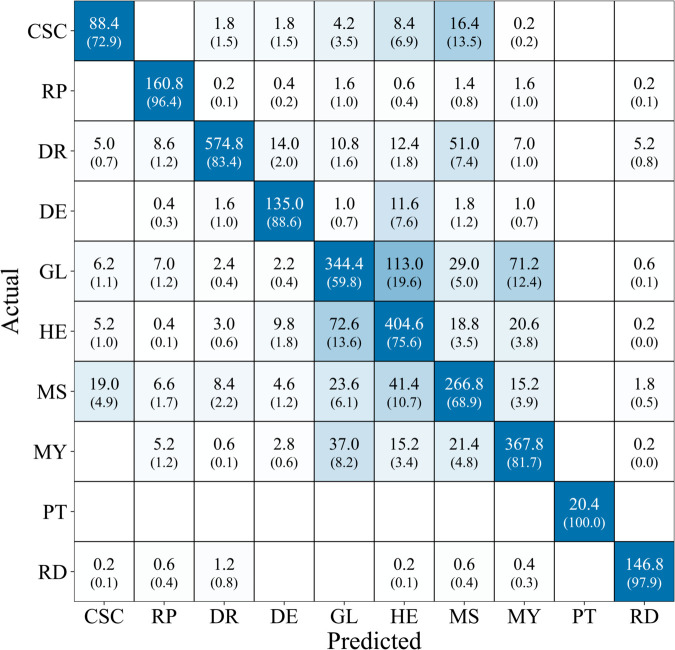
Averaged confusion matrix of the proposed model across all five folds. Each cell reports the mean number of predictions per fold (absolute counts) with percentages in parentheses (normalized to the ground-truth samples per class). The diagonal highlights correct classifications.

Fig 4 shows the averaged confusion matrix of the proposed model across all five folds, denoting absolute counts and percentage values relative to the number of ground-truth samples per class. Strong diagonal values indicate high classification accuracy for several distinct conditions, demonstrating the high performance of the model in correctly identifying retinal pathologies.

For *Central Serous Chorioretinopathy*, 72.9 % of the cases were identified as such, with the most common error being *Central Serous Chorioretinopathy* classified as *Macular Scar* (13.5 % of true *CSC* cases, 16.4 samples per fold on average). *Retinitis Pigmentosa* was correctly identified with high performance: 96.4 % of true cases were classified as true (160.8 samples per fold on average). The most common error was a misclassification as *Glaucoma* or as *Myopia* with 1.6 errors per fold on average, respectively. For *Diabetic Retinopathy*, 83.4 % of the cases were detected. The errors are distributed mainly equally, only the misclassification of *DR* as *Macular Scar* was particularly high with 7.4 % of true *DR* cases. 88.6 % of the ground truth *Disc Edema* were correctly classified, with the most common error being a misclassification as *Healthy* cases (7.6 % of ground truth cases). *Glaucoma* exhibited the lowest classification accuracy: 59.8 % of images labeled as *Glaucoma* were classified into this class (344.4 images on average per fold). Two common errors can be observed: misclassification as *Healthy* (19.6 % of ground truth) and as *Myopia* (12.4 % of ground truth). Regarding *Healthy* samples, 75.6 % were correctly classified. The most common faulty diagnosis was Glaucoma (13.6 % of true healthy samples), followed by *Myopia* and *Macular Scar* (3.8 % and 3.5 %, respectively). 68.9 % of samples exhibiting a *Macular Scar* were truly seen as such, the model classified 10.7 % of those images as *Healthy* and 6.1 % as *Glaucoma*. Our approach demonstrated a higher classification accuracy of 81.7 % for *Myopia*, the most common error was a misclassification as *Glaucoma*. The model achieved perfect (100 %) classification accuracy for *Pterygium* (PT, 102 samples) and near perfect accuracy (97.9 % of true PT samples) for *Retinal Detachment* (RD, 750 samples). For the latter one, the dominant misclassification was Retinal Detachment classified as *Diabetic Retinopathy* (1.2 misclassifications per fold, 0.8 % of true RD samples).

**Fig 5:**
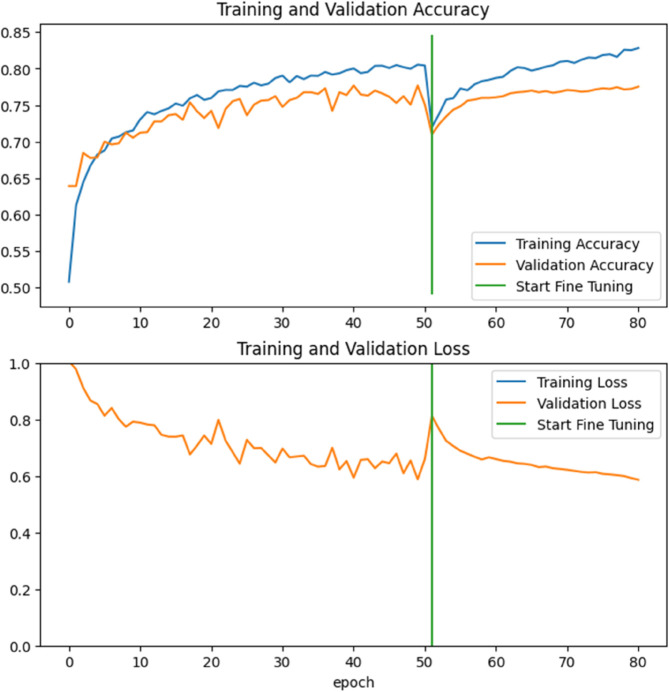
Training dynamics of the proposed model for Fold 0. The upper panel shows the training and validation accuracy, the lower panel shows training and validation loss over 80 epochs. The blue curves correspond to training, the orange curves to validation. The green vertical line marks the start of fine-tuning of the pretrained backbone, after which both accuracy improves and loss decreases. Training and validation curves follow a similar trend, indicating stable convergence without clear signs of overfitting.

The graphs in Fig 5 illustrate the accuracy (top) and loss (bottom) for training (blue) and validation (orange) for Fold 0 before and after the start of the fine tuning (green). The training accuracy increases steadily throughout, while the validation accuracy follows closely, indicating a high generalization. In the loss curves, both training and validation loss decrease rapidly during the initial epochs and stabilize at lower values, suggesting effective learning with minimal overfitting. The green vertical line marks the start of fine-tuning, after which a brief dip is observed, followed by continued performance improvement.

### Explainability of our model

**Fig 6:**
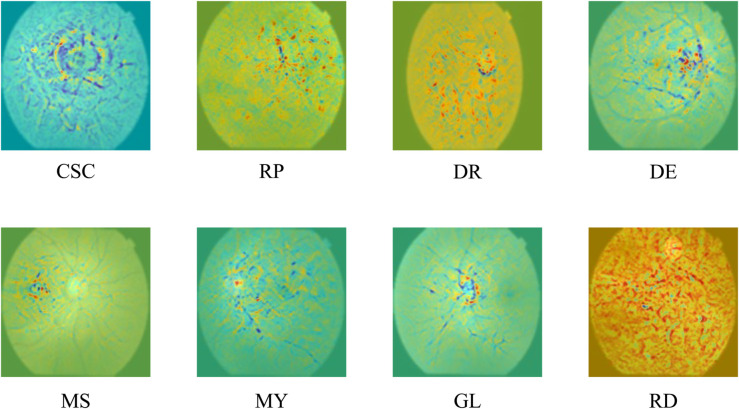
Visualization of pixel-importance maps for eight retinal disease classes (CSC, RP, DR, DE, MS, MY, GL, RD). Warmer colors (red/yellow) indicate image regions with higher relevance for the model’s decision, while cooler colors (blue/green) represent less influence. Healthy and Pterygium are excluded, as they lack typical fundus anomalies or differ in image characteristics [4].

The explainability of deep learning models is particularly crucial in the medical context. On the one hand, this is to increase trust in these systems and, on the other hand, to strengthen the role of such models in decision support, not just in pure diagnostics. For this reason, we also applied an explainability approach to our proposed model. To achieve this, the significance of the individual pixels in the respective images for the respective prediction was visualized. This visualization is shown in Fig 6 below for eight diseases. A visualization of the healthy samples is not useful, as healthy fundus images are characterized by the absence of anomalies. Visualization of the *Pterygium* class is also not useful, as this disease is represented in this dataset by a completely different image type, images of the outer retina of the eye (see Fig 3).

The visualization shows that the model correctly bases its predictions on medically relevant characteristics across all clinical pictures. For *Central Serous Chorioretinopathy*, the model accurately takes into account the area around the rounded serous retinal detachment, and for *Retinitis Pigmentosa*, it takes into account the dark clump pigmentations located in the upper right area in the ground truth. The microaneurysms of the blood vessels typical of *Diabetic Retinopathy*, distributed across the retina, are correctly weighted highly for the respective model prediction. For *Disc Edema*, the typical change in the papilla is given special consideration (displayed on the right side of the fundus image).

The *Macular Scar* located in the left part of the fundus in the sample under consideration is particularly important in the respective prediction. Equally correct, the tilted disc characteristic of *Myopia* is weighted heavily for the correct model prediction. The enlarged cup-to-disc ratio, typical of *Glaucoma*, is correctly taken into consideration by the model. For *Retinal Detachment* (in this sample largely spread), the attention accurately extends across the entire raised retina.

## Discussion

### Analyzing misclassifications

The results of this study demonstrate that leveraging domain-specific features of eye diseases yields benchmarking performance in multiclass classification based on fundus images. The average accuracy of 77.26 % and a balanced accuracy of 82.52 % indicate that the model performs consistently well across all classes, despite class imbalances in the dataset. Nevertheless, an analysis of the model’s misclassifications can be performed.

*Pterygium* was classified perfectly by our model. This observation was to be expected: While other classes in the dataset show retinal fundus images, the *Pterygium* class depicts photographs taken from outside the cornea, which are visually distinct (see Fig 3). However, the correct recognition of all *Pterygium* samples can be interpreted as a sign of good model generalizability, since it correctly identifies this change in perspective even though there are only 102 samples. At the same time, however, this should not be over-interpreted, as only a few predictions per fold are made for *Pterygium* due to the dataset imbalance.

*Retinitis Pigmentosa* and *Retinal Detachment* were recognized with similarly high quality. With 834 and 750 images, respectively, they are also among the more underrepresented classes. Our model thus exhibits behavior that is particularly important for medicine: Since only a small number of samples are available for rare clinical pictures, but these in particular must be reliably recognized, good performance is also important for underrepresented classes.

The dominant number of misclassifications is found in the *Glaucoma*, *Macular Scar* and *Myopia* group and in the differentiation of healthy samples, revealing systematic errors occurring in certain classes and not randomly. This demonstrates the future potential of our model: feature extraction and recognition are present, classes can be differentiated in general, and only certain ones are confused. Such clearly defined error patterns can be reduced by further, targeted model improvements, e.g., through additional training data or a focus on important areas of classes that are difficult to differentiate, made possible by hybrid models [62].

### Contextualization within existing literature

The proposed approach must be situated within the broader landscape of automated eye disease classification research, particularly in light of the dataset quality and their coverage of relevant eye diseases; see Table 1.

The largest number of relevant studies focuses only on a limited number of eye diseases and does not use a peer-reviewed dataset [10–13]. Although some of these studies achieve very high performance, the basic setting does not reflect the complexity of reality, and the data basis is not ideally methodologically robust. Three studies use a peer-reviewed dataset: Grassmann et al. [14], Tomar et al. [16], and Chea/Nam [17]. However, these studies address different degrees of severity of AMD, or few classes, respectively, and thus do not ideally reflect the complexity either. Only He et al. [18] and Al-Fahdawi et al. [19] use seven and eight classes, respectively, in their evaluations, thus covering a broader range, but their dataset is not peer-reviewed. The present model distinguishes between nine diseases, both among themselves and from healthy samples, thus filling this gap. With a balanced accuracy of 82.52 %, it is the first study to address this gap, setting a new reference point. However, due to the imbalance of the dataset and other restrictions of the dataset acknowledged , the generalizability of our results remains limited to the used dataset.

The peer review of the dataset is a necessary condition for a methodologically high-quality study. By using the dataset by Sharmin et al. [4], that is publicly accessible, labeled by domain experts, and encompasses both anterior and posterior eye diseases, our study fulfills this need. Simultaneously, the limits of the dataset used (e.g., size, class imbalance) should be acknowledged when interpreting our results. However, there are different perspectives on the number of classes: On the one hand, it is possible to train a larger set of expert models that, for example, only make a binary distinction between healthy and sick and then progressively narrow down the potential disease in binary decisions. On the other hand, a multiclass approach can be used directly. However, since such an expert tree potentially leads to the propagation of errors (incorrect decisions in, for example, the first binary classification lead to irreversible consequences), these are not preferable. Furthermore, extracted features are not shared, but only created and used within the expert model. It is therefore essential to include as wide a range of diseases as possible, which is done methodically and robustly by the present model [63].

### Consequences of error types

Understanding the clinical implications of false positives and false negatives is essential when evaluating diagnostic models, as not all errors carry the same consequences for patient outcomes and healthcare practice.

In clinical settings, **false positives** in the detection of eye disease can cause unnecessary stress for patients and the risk of additional diagnostic procedures [2,3,64]. While less critical than missed diagnoses, false alarms still contribute to inefficiencies and unnecessary healthcare utilization [1,65]. Our model maintains a low false positive rate, reflected in the high precision (77.26 %), meaning that over three-quarters of "positive" classifications were correct. However, since *Healthy* was integrated as a class on the same hierarchical level as various diseases, "positive" does not refer to a generally "ill" patient, but to a correctly classified disease.

In contrast, **false negatives** pose a more severe risk. Missed diagnoses in eye diseases such as diabetic retinopathy or glaucoma can result in irreversible damage or blindness if left untreated [1,2]. This applies both to samples that are incorrectly classified as healthy, which are therefore denied treatment, and to patients who receive the wrong treatment due to a false negative. The observed false negative rate is comparatively low, demonstrated by the high recall (77.26 %) and F1-Score (77.26 %), which strike a favorable balance between recall and precision.

The integration of the Gaussian filter plays a key role by suppressing misleading activations and enhancing subtle pathological features, thereby improving detection accuracy and reducing over-detection. This contributes both to more reliable classification and to the clinical safety of the model.

### Medical implications

Besides merely interpreting error patterns, our results can be interpreted in light of the visual features typically seen in fundus photography and their diagnostic distinctiveness. This allows a better understanding of the possible reasons behind the misclassifications. *Retinitis Pigmentosa*, *Disc Edema* and *Retinal Detachment* are classified with high accuracies higher than 88 %. These diseases are characterized by distinctive retinal fundus changes:

- *Retinitis Pigmentosa* exhibits characteristic peripheral bone-spicule pigmentation and vessel attenuation [34].
- *Disc Edema* presents with blurred disc margins and often elevated optic discs, making it visually unique [28].
- *Retinal Detachment* shows clear separation of retinal layers and folds, which are rarely confused with other conditions [33].

Due to those distinctive features, high recalls are possible for models that utilize those specific features. Other classes also achieved high classification accuracy, but significantly lower. 83.4 % of *Diabetic Retinopathy* and 81.7 % of *Myopia* were correctly detected as such.

- *Diabetic Retinopathy* presents with recognizable features like microaneurysms, dot-blot hemorrhages, exudates and abnormal blood vessels [1,4,24], especially in moderate to severe stages. These lesions are distributed across the retina, making DR distinct from more localized pathologies [1,24]. However, the features are more subtile than those of conditions presented before.
- *Myopia* shows diffuse and subtle features, such as peripapillary atrophy or general fundus thinning [31], which can visually overlap with other illnesses.

The features of those diseases have been detected with a high, however not highest recall. The lowest performing classes were *Glaucoma*, *Macular Scar* and *Central Serous Chorioretinopathy* with a recall of 59.8 % / 68.9 % and 72.9 %, respectively.

- *Central Serous Chorioretinopathy* is often misclassified as *Myopia* or *Healthy*. It typically shows serous detachment of the neurosensory retina, which can be subtle and may mimic the visual smoothness of a healthy fundus, especially in early stages [4]. In addition, both *CSC* and *Myopia* may present with a slightly thinned or stretched retina, leading to confusion in image-based analysis [27].
- *Macular Scar* is frequently confused with Healthy or DR. Scars may be misinterpreted as exudates or old hemorrhages, particularly if pigmentation or atrophy is mild. Furthermore, macular scars lack active vascular anomalies, which could lead the model to treat them as non-pathological in the absence of strong contrast [30].
- *Glaucoma* usually manifests itself in the form of disc cupping, which is characterized by an enlarged cup-to-disc ratio. These changes are subtle, especially in the early stages [29].

### Practical implications

When it comes to medical issues, research must always be viewed against the backdrop of practical applicability. From a theoretical perspective, to the best of our knowledge, the model we present is the first model capable of classifying a wide range of diseases and healthy samples based on peer-reviewed data while achieving high accuracy, this capability must therefore be made usable in practical deployment. This opens up a variety of possibilities:

For existing medical facilities, such a model can serve as a decision-making aid [66], more than previous models with limited detectable classes. Due to the broad scope of the model and its ability to reliably detect even lesser-known diseases, it is ideally suited to support existing optical diagnostic devices. It can be used not only for final diagnosis, but also for preliminary examinations not performed by doctors, where the model can prioritize or categorize cases.

Thanks to its ability to provide at least a preliminary assessment without the involvement of a doctor, our proposed model can be used in broader screening initiatives for the first time. One conceivable application, for example, is deployment at opticians, which could relieve the burden on doctors’ offices and contribute to improving public health—a capability made possible by the first-ever proven ability to differentiate between various eye diseases based on methodologically robust data sets. Beyond active screening initiatives, high-resolution fundus images are increasingly captured in optometric practices, eye-tracking systems, and even consumer-grade wearable devices. Even if those images do not reach medical-grade quality, combining such systems with the proposed model potentially leads to identifying pathological changes before they manifest as clinical symptoms. This positions the model as a valuable component in preventive healthcare strategies [65,67].

## Conclusion

Eye diseases remain a global health burden and can lead to impairments ranging from visual impairment to blindness [1]. Although deep learning systems are particularly well-suited to their diagnosis, no approach has yet been presented that has classified a wide range of eye diseases using a methodologically robust, peer-reviewed dataset. This work presents a DL-based classification system for nine clinically distinct eye diseases and healthy samples, leveraging the specific characteristics of fundus images by incorporating a Gaussian preprocessing filter and a ResNet-50 backbone via transfer learning. Using a publicly available peer-reviewed dataset and applying 5-fold cross-validation, the proposed method sets a new benchmark in multiclass ophthalmic disease classification by reaching an avg. balanced accuracy of 82.52 %. The results demonstrate that diagnostic performance can be significantly improved not solely through computationally more expensive architectures, but through tailored image preprocessing. Tools such as the one developed in this study hold promise for addressing diagnostic bottlenecks, particularly in low-resource or remote settings. By offering an accurate and validated solution for multiclass disease classification, this work aligns with the broader public health imperative to reduce preventable blindness through early detection and intervention.

### Limitations

Despite its contributions, this study faces several limitations.

**First**, the model was trained exclusively on color fundus photographs. While these images are widely used in ophthalmology, they provide only a two-dimensional view of retinal structures which may reduce the model’s ability to detect subtle structural anomalies. Additionally, the fundus images used have a relatively small angle. Modern fundus cameras can achieve broader images of up to 180^°^ [13], enabling a more robust classification of eye diseases. **Second**, the dataset consists of color fundus images of sufficient quality for analysis. However, in practice, image degradation can occur due to factors such as patient movement, poor exposure, or equipment limitations, potentially affecting model performance. To validate the robustness of our model, additional suboptimal quality image data would be required, which were not available for this study. **Third**, the dataset used, while peer-reviewed and clinically annotated, exhibited significant class imbalance. Some categories in particular were significantly underrepresented: With 102 samples, the *Pterygium* class is by far the least common class, accounting for only about 0.63 % of the dataset. Such a strong imbalance can potentially influence training, even if countermeasures were taken by using class weights. This also applies to other underrepresented classes such as *CSC* (606 samples, 3.73 % of the dataset) or *RD* (750 samples, 4.62 % of the dataset), which the model could prefer over more frequently represented classes in its predictions due to their low representation. Such a limitation could limit generalizability in real-world screening scenarios. **Fourth**, whereas we used a large dataset for this research problem, it does not fully reflect all variations of eye conditions. Underrepresentation of certain groups could affect model performance between subpopulations. Addressing these biases would require extensive data. **Last**, the accuracy of deep learning models depends on the quality of the labeled data. Since our dataset is labeled based on human diagnoses, mislabeling due to errors is possible, affecting model performance. Correcting this would require expert re-evaluation, which was beyond the scope of this study.

### Future work

Building upon the findings and identified limitations of this study, future work should aim to extend the methodological, clinical, and translational scope of the proposed system. **First**, incorporating additional imaging modalities such as optical coherence tomography, fundus autofluorescence, or fluorescein angiography could improve the system’s ability to detect structural and functional anomalies not visible in two-dimensional fundus photographs alone. This multimodal fusion may enhance sensitivity for early or subtle manifestations of diseases like glaucoma or macular degeneration, where cross-sectional information is critical. **Second**, future research should address dataset limitations by curating more balanced and comprehensive datasets that reflect the true prevalence and diversity of ophthalmic conditions, particularly underrepresented classes like Pterygium or CSCR. Synthetic augmentation strategies or targeted data collection efforts could support this goal. **Third**, to assess real-world applicability, the model should be tested in prospective deployment scenarios such as community screening programs, mobile eye care units, and teleophthalmology platforms. These studies should evaluate not only diagnostic performance but also usability, robustness under variable imaging conditions, and integration with existing clinical workflows.

In summary, our model proves that a methodologically robust classification of a wide range of eye diseases is possible when the specific characteristics of such fundus images are leveraged. It thus contributes directly to the advancement of such systems and, consequently, to the improvement of individual diagnoses and public health.
